# Comparison and Prognostic Analysis of Adjuvant Radiotherapy versus Salvage Radiotherapy for Treatment of Radically Resected Locally Advanced Esophageal Squamous Cell Carcinoma

**DOI:** 10.1155/2016/8548694

**Published:** 2016-10-16

**Authors:** Xin Xu, Hua-Ying Xie, Di Zhou, Ren-Hua Huang, Yong-Rui Bai, Jun Yuan, Ming Ye

**Affiliations:** ^1^Department of Radiation Oncology, Renji Hospital, School of Medicine, Shanghai Jiao Tong University, Shanghai 200127, China; ^2^Department of Radiation Oncology, Dongfang Hospital Affiliated to Tongji University, Shanghai 200120, China

## Abstract

*Objective*. To compare adjuvant radiotherapy and salvage radiotherapy after radical resection for treatment of esophageal squamous cell carcinoma (ESCC).* Methods*. Data from 155 patients with locally advanced ESCC who underwent radical resection and received postoperative radiotherapy from 2005 to 2011 were reviewed. Seventy-nine patients received adjuvant radiotherapy and 76 received salvage radiotherapy after locoregional recurrence.* Results*. The median disease-free survival (DFS) and overall survival (OS) were significantly higher in the adjuvant radiotherapy group than the salvage radiotherapy group (DFS 25.73 months versus 10.73 months, *P* < 0.001; OS 33.33 months versus 26.22 months, *P* = 0.006). The independent prognostic factors for DFS were performance status (PS) before radiotherapy and pathological stage in the adjuvant radiotherapy group, compared with lymph node metastasis, tumor location, and adjuvant chemotherapy in the salvage radiotherapy group. The independent prognostic factors for OS were age and PS in both groups. No differences in median DFS and OS between the groups were observed in patients aged > 65 years or with PS ≥ 2.* Conclusion*. Compared to salvage radiotherapy, postoperative adjuvant radiotherapy can prolong DFS and OS for patients with radically resected local advanced ESCC but cannot improve survival for patients aged > 65 years or with PS ≥ 2.

## 1. Introduction

Esophageal cancer was the third most common malignant tumor in males and the fifth most common in females, in China in 2015 [1]. Compared to Western countries, the incidence of esophageal cancer in China is significantly higher, with 477,900 new cases diagnosed each year, and ~375,000 people die from the disease annually [2]. The most common pathological type of esophageal cancer in China is squamous cell carcinoma (SCC). Although neoadjuvant chemoradiotherapy (CRT) followed by esophagectomy is currently the recognized standard treatment for patients with locally advanced esophageal SCC (ESCC), many patients still choose surgery as their initial therapy. However, locoregional recurrence remains a major cause of treatment failure and develops in 40–60% of patients [3].

In 2014, a surveillance, epidemiology, and end results (SEER) database analysis of patients with locally advanced ESCC showed that bimodal therapy (i.e., surgery with radiotherapy) improved cancer specific survival and overall survival (OS) compared to unimodal therapy [4]. However, no differences were seen between patients undergoing preoperative or postoperative radiotherapy [4]. Postoperative radiotherapy has the advantage of selecting patients based on pathological findings; however, there are issues with patient tolerability after surgery and difficulties in target volume delineation (as the gross tumor has been removed and the anatomy has changed). Indeed, the relative lack of large randomized clinical trials (RCTs) has limited our ability to draw firm conclusions around the benefits of postoperative radiotherapy in patients with ESCC, especially in OS [5]. On the other hand, the treatment methods are limited once the tumor relapses, and few patients are given the opportunity for a second operation. Salvage radiation might be an effective treatment strategy. But for the patients who have received adjuvant radiotherapy after surgery, a high dose radiotherapy will be difficultly performed when the tumor recurred, because of the dose limitation of the normal tissues. Since the role of adjuvant radiotherapy is indeterminate, shall we choose radiation till the tumor relapsed as a salvage treatment approach? However, few studies have directly compared adjuvant and salvage radiotherapies after radical resection in patients with ESCC. While Yamashita et al. [6] indicated that the prognosis of patients who received salvage radiotherapy for postoperative locoregional recurrence of esophageal cancer was comparable with patients receiving planned postoperative radiotherapy after esophagectomy, the number of cases included in this study was small (*n* = 16). With the increasing focus on neoadjuvant CRT, it is unlikely that further studies examining the role of adjuvant CRT will ever be conducted.

Therefore, in the present study, we performed a retrospective analysis of patients with ESCC that underwent postoperative adjuvant radiotherapy compared to those who underwent salvage radiotherapy for locoregional recurrence. The aim of this study was to evaluate the value of adjuvant radiotherapy in patients with ESCC who have undergone radical resection and the related prognostic factors.

## 2. Patients and Methods

### 2.1. Patients

From January 2005 to December 2011, 155 patients with pathologically proven ESCC, who received radiotherapy at the Department of Radiation Oncology, Renji Hospital, School of Medicine, Shanghai Jiaotong University, after radical resection, were retrospectively reviewed. In total, 79 of these patients received adjuvant radiotherapy in the first 3 months after operation, and the other 76 patients received radiotherapy for local or regional recurrence. All patients underwent radical resection and lymph node dissection. Patients with palliative resection and tumor residual were excluded. The salvage radiotherapy group included patients with esophageal local recurrence and regional lymph node recurrence. Esophageal local recurrence was proven by pathology (endoscopic biopsy), while regional lymph node recurrence was proven by enhanced computed tomography (CT), PET/CT, or lymph node biopsy. Tumors were staged based on the TNM classification of the American Joint Committee on Cancer (AJCC) in 2010.

### 2.2. Radiotherapy

Adjuvant radiotherapy using three-dimensional conformal radiotherapy (3D-CRT) was administered 4–12 weeks postoperatively. The extent of the irradiation field was determined based on the primary site in the esophagus. The radiotherapy area contained the bilateral supraclavicular area, mediastinum, and subcarinal area for lesions in the upper thoracic segment of the esophagus. The superior border of the middle thoracic segment was the upper edge of the first thoracic vertebra; and the upper boundary of the lower thoracic segment was 3 cm above the upper edge of the gross tumor identified on preoperative computed CT images. The inferior border of the midlower thoracic segment was 3-4 cm below the lower edge of the gross tumor, as identified on preoperative CT images. The field included the related drainage areas of the mediastinal lymph nodes and the primary esophageal tumor bed. The total dose was 50 Gy in 25 fractions within 5 weeks.

Both 3D-CRT and intensity-modulated radiotherapy (IMRT) were used for patients undergoing salvage radiotherapy. The gross tumor volume (GTV) included all known gross disease, as determined by the imaging and endoscopic findings. The clinical target volume (CTV) was defined as the GTV plus 3 cm longitudinal margins and 0.8 cm radial margins and included the correlated lymphatic drainage regions. The planning target volume (PTV) was defined as the CTV plus a 0.5 cm margin in all directions. These areas were irradiated with 40–50 Gy in 20–25 fractions, and then the dose was boosted to 60–70 Gy for the GTV only. The median irradiation dose was 60 Gy (range 50.4–70 Gy). The dose constraint for the spinal cord was a maximum dose of <45 Gy. For lungs, the mean dose and V20 were limited within 15 Gy and 30%, respectively.

### 2.3. Statistical Analysis

Statistical analysis was performed using SPSS 21.0 software (SPSS Inc., Chicago, IL, USA). OS time was calculated from the date of operation to the date of death or the most recent follow-up time (September 1, 2015). DFS time is defined as survival without disease progression from the date of operation. The chi-squared test was used to compare differences in clinicopathological features between the two groups. Median OS and DFS were estimated using Kaplan-Meier curves. Univariate analysis and multivariate analysis were performed to investigate prognostic factors by the log-rank test and the Cox regression model. A *P* value < 0.05 was considered statistically significant.

## 3. Results

### 3.1. Patients' Characteristics

The patients' characteristics are presented in Table 1. There were no statistical differences in age, gender, surgical-pathological stage, lymph node metastasis, tumor location, and adjuvant chemotherapy after operation between the two treatment groups. However, patients in the salvage radiotherapy group had poorer PS before radiotherapy and more patients had more tumors in the pT1 and pT2 stage.

### 3.2. Survival

**Figure 1:**
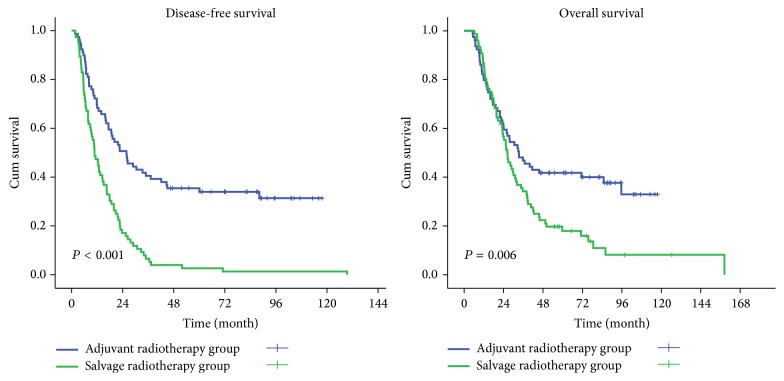
Disease-free survival (DFS) and overall survival (OS) of patients in the two treatment groups.

During follow-up, 116 patients died: 49 patients in the adjuvant radiotherapy group and 67 in the salvage radiotherapy group. The median follow-up time was 76.9 months for the surviving patients. The adjuvant radiotherapy group had significantly improved OS compared to the salvage radiotherapy group. The median OS for cases in the adjuvant radiotherapy group was 33.3 months (95% CI: 21.43–45.23 months), compared to 26.2 months (95% CI: 22.10–30.16 months) for cases in the salvage radiotherapy group (*P* = 0.006). The 2-year and 5-year OS rate were 69.6% and 41.8% in the adjuvant radiotherapy group, respectively, compared with 55.3% and 17.9% in the salvage radiotherapy group (*P* = 0.046 and *P* < 0.01, resp.). However, there was no difference in the 1-year OS rate between the two groups (79.7% versus 84.2%, *P* = 0.30; Figure 1).

In patients in the adjuvant radiotherapy group, disease progressed in 41 cases. There were 15 (36.6%) cases of locoregional recurrence, 22 (53.7%) cases of distant metastases, and 4 (9.7%) cases of simultaneous locoregional recurrence and distant metastases in the adjuvant radiotherapy group. Twelve patients had no evidence of disease progression before they died, including one patient who died from an accident, one who died of severe pneumonia, two who died of upper gastrointestinal hemorrhage, four who died of dyscrasia, and four who died from unknown causes. The salvage radiotherapy group included 10 (13.2%) patients with esophageal recurrence, 59 (77.6%) patients with regional lymph node recurrence, and 7 (9.2%) patients with simultaneous local and regional lymph node recurrence. Of these 76 patients, 17 (22.4%) patients also had distant metastases at the same time. The median DFS in the adjuvant radiotherapy group was significantly prolonged compared to the salvage radiotherapy group (25.7 months versus 10.7 months, *P* < 0.001). The 1-year, 2-year, and 5-year DFS rates in the adjuvant radiotherapy group were 68.4%, 50.6%, and 34.0%, respectively, compared to 47.4%, 17.1%, and 2.6% in the salvage radiotherapy group (*P* = 0.06, *P* < 0.01, and *P* < 0.01; Figure 1). The median survival time in the salvage radiotherapy group after radiotherapy was 9.6 months. Median OS in this group was 13.1 months in patients without distant metastases compared to 6.9 months in patients with distant metastases (*P* = 0.03).

### 3.3. Prognostic Factors and Subgroup Analysis

On univariate analysis, OS was significantly associated with age, PS, and surgical-pathological stage in the adjuvant radiotherapy group, while it was only associated with age and PS in the salvage radiotherapy group (*P* < 0.05). Multivariate analysis showed that age and PS were the independent prognostic factors for OS in both groups. Moreover, DFS was significantly associated with PS and surgical-pathological stage in the adjuvant radiotherapy group on univariate analysis, while it was associated with gender, lymph node metastasis, tumor location, and adjuvant chemotherapy after operation in the salvage radiotherapy group (*P* < 0.05). Multivariate analysis showed that PS and surgical-pathological stage were independent prognostic factors for DFS in the adjuvant radiotherapy group, while lymph node metastasis, tumor location, and adjuvant chemotherapy after operation were independent prognostic factors in the salvage radiotherapy group (Table 2).

**Figure 2:**
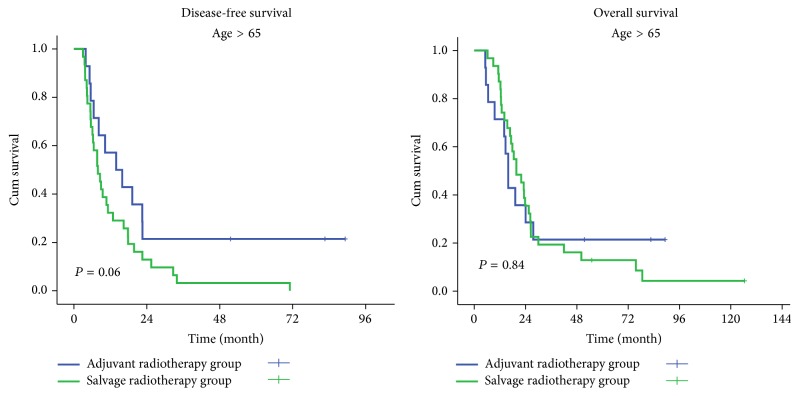
Disease-free survival (DFS) and overall survival (OS) of patients aged > 65 years in the two treatment groups.

**Figure 3:**
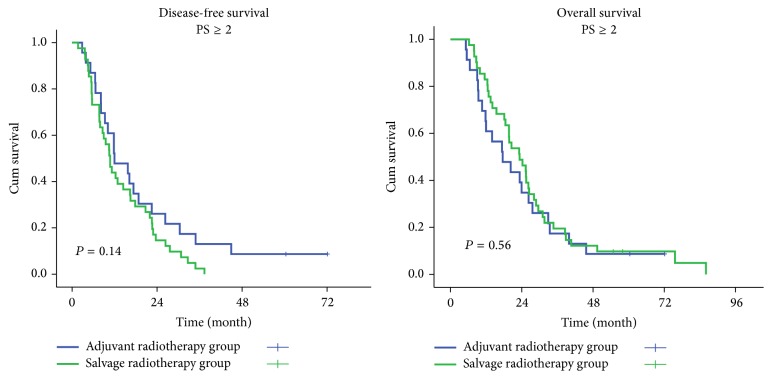
Disease-free survival (DFS) and overall survival (OS) of patients with performance status (PS) ≥ 2 in the two treatment groups.

All patients were stratified by gender, age, PS, surgical-pathological stage, pT stage, pN status, histological grade, tumor location, and adjuvant chemotherapy. The OS in adjuvant radiotherapy group was significantly improved in patients who were male, aged ≤ 65 years, with a PS < 2, of surgical-pathological stage II and pT2 stage, and with adjuvant chemotherapy, compared with the salvage radiotherapy group (*P* < 0.05). No significant differences in OS were observed between patients in other subgroups. The adjuvant radiotherapy improved DFS significantly in almost all the subgroups, except the patients who were aged > 65 years, with a PS ≥ 2, and with a pT4 stage (Table 2, Figures 2 and 3).

## 4. Discussion

In this study, we confirmed that patients who underwent planned adjuvant radiotherapy after surgery had better OS and DFS compared to patients who underwent salvage radiotherapy after recurrence. While the 2-year and 5-year OS rates were significantly different between the two groups, the 1-year OS rates were similar. This implies that the improvement of adjuvant radiotherapy for OS might be limited to the long-term survival rate and not the short-term survival rate. A potential explanation may be the poor nutritional status and weakened immunity in patients after invasive surgery, which is exacerbated by the radiotherapy. Indeed, we found 37.5% (6/16) of patients who lived for less than 1 year died without disease progression, while this rate was much lower (18.2%, 6/33) in patients who lived for more than 1 year.

Our study also showed that the patients who underwent salvage radiotherapy still had a median OS time of 9.6 months, which increased to 13.1 months in patients who had locoregional recurrence without simultaneous distant metastasis. Indeed, similar to our findings, some recent studies report that the median OS of salvage radiotherapy for locoregional recurrence after curative surgery can reach 9–21 months [3, 6, 7]. Therefore, once the tumor relapses, salvage radiation with a radical dose might be a promising treatment strategy for improving tumor remission and survival.

To determine the prognostic factors, we performed univariate and multivariate analyses. We found that PS and age were independent prognostic factors for OS in both the adjuvant radiotherapy group and the salvage radiotherapy group. PS and surgical-pathological stage were independent prognostic factors for DFS in the adjuvant radiotherapy group. On the other hand, lymph node metastasis, tumor location, and adjuvant chemotherapy after operation were independent prognostic factors for DFS in the salvage radiotherapy group. These results imply that the OS tends to be associated with factors related to the patients' general condition, for example, PS and age, while DFS tends to depend on factors related to the disease condition, such as pathological stage, lymph node metastasis, tumor location, and adjuvant chemotherapy. However, we did not find that the OS and DFS were related to pT stage and histological grade in either univariate or multivariate analyses, which is inconsistent with the historical results [8–10]. This difference in our results compared to previous studies might be due to the small sample size of some our subgroups, such as the pT1, pT4, histological grade 1, and histological grade 3 subgroups.

While radiotherapy has been widely used in adjuvant treatment to prevent locoregional recurrence in patients with ESCC, the indication for this treatment approach is undefined. In the past, patient selection for adjuvant radiotherapy has almost always been based on clinical histopathological staging, with controversial findings. Some studies found that adjuvant radiation was associated with increased survival only in patients with stage III ESCC and in those who were lymph node positive (especially with extracapsular lymph node extension) [11–15]. On the other hand, other researches suggest that patients with resectable thoracic ESCC may not benefit from postoperative adjuvant therapy [16, 17]. For example, when Chen et al. [16] evaluated the value of postoperative adjuvant therapy for resectable thoracic ESCC, the 5-year DFS and OS rates were similar in the surgery-alone and adjuvant therapy groups. Furthermore, in their subgroup analysis, they found that patients with an N0 tumor stage who underwent surgery alone had a higher 5-year DFS than those undergoing postoperative adjuvant therapy [16]. In another study, patients were stratified based on their T stage, and postoperative radiotherapy was found to boost the survival and reduce the relapse rate of tumors in patients at the pT4a tumor stage [17]. Finally, Wang et al. [18] indicated that postoperative adjuvant therapy did not affect OS and DFS of patients with pT3N0M0 stage thoracic ESCC. However, in patients with early stage pT2N0M0 ESCC, those with a high expression of Ku80 had a worse OS and DFS, and adjuvant radiotherapy could significantly improve survival in these patients [19]. Based on these previous studies, we hypothesized that only certain patients may benefit from adjuvant radiotherapy but that selecting patients based only on their histopathological stage may be insufficient.

To evaluate the role of radiotherapy in patients with different characteristics and the influence of different prognostic factors, we performed the subgroup analysis. Our subgroup analysis also showed that, compared to salvage radiotherapy, adjuvant radiotherapy improved both OS and DFS in the following subgroups: male, ≤65 years, PS < 2, stage II, and with adjuvant chemotherapy. However, adjuvant radiotherapy did not improve either OS or DFS in patients aged > 65 years, with a PS ≥ 2, and those at the pT4 stage. We did not observe survival superiority of adjuvant radiotherapy in female patients. Indeed, the survival of female patients was slightly lower than male patients, although the difference was not statistically significant. One possible reason for this result might be that the number of female patients included in our study was small (24 patients) and most (17/24) had a PS ≥ 2. We also observed that adjuvant radiotherapy improved the DFS but not OS in patients with stage III ESCC and patients without adjuvant chemotherapy. Both surgery and radiotherapy are local treatment methods, and although adjuvant radiotherapy may reduce the locoregional recurrence rate in these patients, it is likely that most will eventually develop systemic metastasis leading to death. Therefore, adjuvant radiotherapy might not improve the OS in these patients. In addition, we did not observe the improvement in DFS and OS in the adjuvant radiotherapy group for patients aged over 65 years and with a PS ≥ 2. This indicates that patients with advanced age and a poor general condition after operation might not benefit from the adjuvant radiotherapy. Therefore, adjuvant radiotherapy should be carefully selected in these patients. This result also suggests that the patient's general condition should be considered when making a decision to administer adjuvant therapy. The same result was also observed in the pT4 subgroup; however, this may be caused by the small sample bias considering there were only three patients at the pT4 stage in the salvage radiotherapy treatment group.

Our study also has some limitations. First, it is a single-institution, retrospective study, and, therefore, its retrospective nature may undermine the power of our study. Second, the relatively small patient numbers in some subgroups may limit its statistical power. Third, the selection of the control group may not have been the optimal choice as we included recurrent cases and excluded those without relapse. Therefore, our results need further validation through a large-scale, prospective study.

## 5. Conclusion

In conclusion, our study showed that postoperative adjuvant radiotherapy was superior in terms of OS and DFS to salvage radiotherapy after locoregional recurrence for the treatment of locally advanced ESCC. However, the improvement in OS appears to be limited to the long-term survival rate. As PS and age were independent prognostic factors for OS, adjuvant radiotherapy should be carefully selected in patients with advanced age and poor general condition.

## Figures and Tables

**Table 1:** Patient characteristics in the two treatment groups.

	Adjuvant radiotherapy group	Salvage radiotherapy group	*P* value
*Gender*			
Male, *n* (%)	64 (81.0)	63 (82.9)	0.84
Female, *n* (%)	15 (19.0)	13 (17.1)	
*Age (years), mean (range)*	58 (44–80)	63 (46–82)	
>65 years, *n* (%)	14 (17.7)	22 (28.9)	0.128
≤65 years, *n* (%)	65 (82.3)	54 (71.1)	
*Performance status*			
<2, *n* (%)	56 (70.9)	36 (47.4)	0.003
≥2, *n* (%)	23 (29.1)	40 (52.6)	
*Surgical-pathological stage*			
I, *n* (%)	1 (1.3)	3 (3.9)	0.07
II, *n* (%)	34 (43.0)	44 (57.9)	
III, *n* (%)	44 (55.7)	29 (38.2)	
*pT stage*			
1, *n* (%)	0 (0.0)	4 (5.3)	0.01
2, *n* (%)	14 (17.7)	23 (30.3)	
3, *n* (%)	53 (67.1)	46 (60.5)	
4, *n* (%)	12 (15.2)	3 (3.9)	
*pN status*			
−, *n* (%)	38 (8.1)	38 (50.0)	1.0
+, *n* (%)	41 (51.9)	38 (50.0)	
*Histologic grade*			
1, *n* (%)	8 (10.1)	4 (5.3)	0.61
2, *n* (%)	51 (64.6)	48 (63.2)	
3, *n* (%)	11 (13.9)	14 (18.4)	
Unknown, *n* (%)	9 (11.4)	10 (13.2)	
*Tumor location*			
Upper, *n* (%)	11 (13.9)	9 (11.8)	0.76
Middle, *n* (%)	54 (68.4)	55 (72.4)	
Lower, *n* (%)	14 (17.7)	12 (15.8)	
*Adjuvant chemotherapy*			
Yes, *n* (%)	35 (44.3)	39 (51.3)	0.07
No, *n* (%)	44 (55.7)	37 (48.7)	

**Table 2:** Univariate and subgroup analysis of overall survival (OS) and disease-free survival (DFS) in the two treatment groups.

	Median OS (months)			Median DFS (months)		
	Adjuvant radiotherapy group	Salvage radiotherapy group	*P* value	Adjuvant radiotherapy group	Salvage radiotherapy group	*P* value
*Gender*						
Male	33.4	26.9	0.02^*∗*^	25.9	12.4	0.00^*∗*^
Female	27.6	25.4	0.20	18.6	7.7	0.01^*∗*^
*P* value	0.85	0.18		0.87	0.04	
*Age*						
>65 years	15.9	19.7	0.84	13.9	7.9	0.06
≤65 years	39.9	32.1	0.03^*∗*^	28.8	13.4	0.00^*∗*^
*P* value	0.02^*∗*^	0.02^*∗*^		0.10	0.10	
*Performance status*						
<2, *n* (%)	84.9	38.0	0.03^*∗*^	37.2	12.4	0.00^*∗*^
≥2, *n* (%)	17.6	23.3	0.56	12.0	10.7	0.14
*P* value	0.00^*∗*^	0.00^*∗*^		0.00^*∗*^	0.46	
*Surgical-pathological stage*						
II	71.4	25.4	0.00^*∗*^	37.2	10.7	0.00^*∗*^
III	23.3	26.4	0.20	13.9	9.2	0.00^*∗*^
*P* value	0.04^*∗*^	0.23		0.02^*∗*^	0.19	
*pT stage*						
2	NA	25.3	0.02^*∗*^	NA	12.4	0.00^*∗*^
3	26.3	26.5	0.07	19.2	9.2	0.00^*∗*^
4	32.5	31.0	0.40	22.5	21.9	0.44
*P* value	0.26	0.81		0.10	0.37	
*pN status*						
−	36.8	31.6	0.06	26.3	14.6	0.00^*∗*^
+	30.6	23.5	0.06	18.7	8.9	0.00^*∗*^
*P* value	0.40	0.06		0.33	0.02^*∗*^	
*Histologic grade*						
1	95.7	11.5	0.05	37.2	3.2	0.01^*∗*^
2	27.6	26.5	0.19	20.1	11.2	0.00^*∗*^
3	NA	23.37	0.05	NA	10.7	0.00^*∗*^
*P* value	0.27	0.93		0.34	0.06	
*Tumor location*						
Upper	62.0	30.0	0.14	NA	5.4	0.00^*∗*^
Middle	30.1	25.3	0.07	20.1	10.7	0.00^*∗*^
Lower	35.2	26.4	0.09	22.57	11.2	0.04^*∗*^
*P* value	0.20	0.40		0.32	0.00^*∗*^	
*Adjuvant chemotherapy*						
Yes	33.43	31.0	0.02^*∗*^	30.4	14.57	0.00^*∗*^
No	26.3	23.3	0.12	22.5	6.9	0.00^*∗*^
*P* value	0.32	0.43		0.90	0.01^*∗*^	

^*∗*^ *P* < 0.05.

NA: the median OS or DFS had not been reached.

## References

[1] Chen W., Zheng R., Baade P. D. (2016). Cancer statistics in China, 2015. *CA: A Cancer Journal for Clinicians*.

[2] Siegel R. L., Miller K. D., Jemal A. (2016). Cancer statistics, 2016. *CA Cancer Journal for Clinicians*.

[3] Ma D.-Y., Tan B.-X., Liu M., Li X.-F., Zhou Y.-Q., Lu Y. (2014). Concurrent three-dimensional conformal radiotherapy and chemotherapy for postoperative recurrence of mediastinal lymph node metastases in patients with esophageal squamous cell carcinoma: a phase 2 single-institution study. *Radiation Oncology*.

[4] Worni M., Castleberry A. W., Gloor B. (2014). Trends and outcomes in the use of surgery and radiation for the treatment of locally advanced esophageal cancer: a propensity score adjusted analysis of the surveillance, epidemiology, and end results registry from 1998 to 2008. *Diseases of the Esophagus*.

[5] Gwynne S., Wijnhoven B. P. L., Hulshof M., Bateman A. (2014). Role of chemoradiotherapy in oesophageal cancer—adjuvant and neoadjuvant therapy. *Clinical Oncology*.

[6] Yamashita H., Nakagawa K., Tago M., Nakamura N., Shiraishi K., Ohtomo K. (2005). Salvage radiotherapy for postoperative loco-regional recurrence of esophageal cancer. *Diseases of the Esophagus*.

[7] Zhang J., Peng F., Li N. (2012). Salvage concurrent radio-chemotherapy for post-operative local recurrence of squamous-cell esophageal cancer. *Radiation Oncology*.

[8] Chen S.-B., Weng H.-R., Wang G. (2016). The impact of adjuvant radiotherapy on radically resected T3 esophageal squamous cell carcinoma. *Journal of Cancer Research and Clinical Oncology*.

[9] Xu Y., Liu J., Du X. (2013). Prognostic impact of postoperative radiation in patients undergoing radical esophagectomy for pathologic lymph node positive esophageal cancer. *Radiation Oncology*.

[10] Chen J., Pan J., Liu J. (2013). Postoperative radiation therapy with or without concurrent chemotherapy for node-positive thoracic esophageal squamous cell carcinoma. *International Journal of Radiation Oncology Biology Physics*.

[11] Xiao Z. F., Yang Z. Y., Liang J. (2003). Value of radiotherapy after radical surgery for esophageal carcinoma: a report of 495 patients. *Annals of Thoracic Surgery*.

[12] Xiao Z.-F., Yang Z.-Y., Miao Y.-J. (2005). Influence of number of metastatic lymph nodes on survival of curative resected thoracic esophageal cancer patients and value of radiotherapy: report of 549 cases. *International Journal of Radiation Oncology Biology Physics*.

[13] Wang Z. W., Luan Z. P., Zhang W. (2014). Postoperative chemoradiotherapy improves survival in esophageal squamous cell cancer with extracapsular lymph node extension. *Neoplasma*.

[14] Shridhar R., Weber J., Hoffe S. E., Almhanna K., Karl R., Meredith K. (2013). Adjuvant radiation therapy and lymphadenectomy in esophageal cancer: a SEER database analysis. *Journal of Gastrointestinal Surgery*.

[15] Schreiber D., Rineer J., Vongtama D. (2010). Impact of postoperative radiation after esophagectomy for esophageal cancer. *Journal of Thoracic Oncology*.

[16] Chen H., Wu Z., Chen J. (2015). Postoperative adjuvant therapy for resectable thoracic esophageal squamous cell carcinoma: a retrospective analysis of 426 cases. *Medical Oncology*.

[17] Chen X., Chen J., Zheng X. (2015). Prognostic factors in patients with thoracic esophageal carcinoma staged pT_1–4a_N_0_M_0_ undergone esophagectomy with three-field lymphadenectomy. *Annals of Translational Medicine*.

[18] Wang Y., Wang L., Yang Q. (2015). Factors on prognosis in patients of stage pT3N0M0 thoracic esophageal squamous cell carcinoma after two-field esophagectomy. *Journal of Cancer Research and Therapeutics*.

[19] Wang S., Wang Z., Yang Z. (2016). Postoperative radiotherapy improves survival in stage pT2N0M0 esophageal squamous cell carcinoma with high risk of poor prognosis. *Annals of Surgical Oncology*.

